# Maternal age, education level and migration: Socioeconomic determinants for smoking during pregnancy in a field study from Turkey

**DOI:** 10.1186/1471-2458-10-325

**Published:** 2010-06-09

**Authors:** Isil Ergin, Hur Hassoy, Feride A Tanik, Gokce Aslan

**Affiliations:** 1 Ege University School of Medicine, Department of Public Health, Bornova, 35100, Izmir, Turkey

## Abstract

**Background:**

Smoking during pregnancy has been associated with socioeconomic determinants and it is recognized as the most important preventable risk factor for an unsuccessful pregnancy outcome. Turkey has national data on the prevalance of smoking during pregnancy; however there is no data on the characteristics of the high-risk population. This is a field study that aims to identify socioeconomic determinants for smoking during pregnancy as well as differentiating the daily and occasional smokers.

**Method:**

Cross sectional study was conducted among women with 0-5 year old children living in the area served by Primary Health Care Center (PHCC) in Burhaniye, Turkey. Face-to-face interviews were conducted by the researchers during January-March 2008 at the home of the participants with 83.7% response rate (n = 256). The relation of "smoking during pregnacy" and "daily smoking during pregnancy" with the independent variables was determined with χ^2 ^tests. Women's age, educational level, number of previous births, place of origin, migration, partner's educational level, poverty, perceived income, social class were evaluated. Statistical significance was achieved when the *p *value was less than 0.05. The variables in relation with the dependent variables in the χ^2 ^tests were included in the forward-stepwise logistic analysis.

**Results:**

Prevalance of smoking during pregnancy was 22.7%. The majority (74.1%) were daily smokers. Young mothers (< 20), low educated women and migrants were at increased risk for smoking during pregnancy. Low education and being a migrant were risk factors for daily consumption (p < 0.05).

**Conclusions:**

Systematic attention should be paid to socioeconomic determinants in smoking for pregnant women, especially in countries like Turkey with high rates of infant and mother mortality and substantial health inequalities. Young mothers (< 20), low educated women and migrants are important groups to focus on.

## Background

Maternal smoking increases perinatal mortality by 150%. It is responsible for 15% of miscarriages and 20-30% of low birthweights [1]. The association between smoking and adverse pregnancy outcomes shows a dose-response relation and thus increases with the amount smoked [2]. Despite improved general knowledge of the hazards, the majority of pregnant smokers continue to smoke, however; 20%-40% of smokers quit during pregnancy [2-4]. There has been a decline in smoking during pregnancy in some countries in the last decades, due primarily to a decrease in smoking initiation [2]. However, smoking prevalance have risen from 18% (1998) to 28% (2003) among young women in Turkey and for pregnant women the smoking prevalance is 15% [5,6].

Smoking was identified as one of the major causes of morbidity and mortality and question has been how this and its inequitable distribution come about. In fact, what are the causes of the causes? This brings up the questioning on the social determinants of cigarette smoking [7]. This behaviour and its social patterning are largely determined by social factors. Smoking during pregnancy has also been associated with socioeconomic determinants [2,3,8,9]. Socioeconomic disadvantage that relates to pregnancy outcome operates through intermediate factors. Cigarette smoking is amongst these factors [10]. Smoking explains up to half of the adverse perinatal outcome among mothers in the lowest socioeconomic groups [11]. Considering this effect, the importance of smoking prevalence of pregnant women among different socioeconomic groups becomes more significant especially for countries like Turkey which have high rates of infant and mother mortality and substantial health inequalities.

However, Turkey has no data on the characteristics of the high-risk population. A better understanding of socioeconomic determinants is essential and will serve to identify the subgroups of the population who need most attention prenatally in policies aiming to reduce smoking.

This data, although not nationally representative, is a population-based sample executed at a town, representing urban and rural settings. This study aims to identify socioeconomic factors for smoking during pregnancy as well as differentiating the daily and occasional smokers.

## Methods

### Setting and Data Collection

Burhaniye is a town at the western coast of Turkey with a population of 43199. This population is engaged in farming, olive oil industry and tourism and so has a combination of characteristics of urban and rural settings where tradition and modernity intersect. The town has two Primary Health Care Centers (PHCC) giving service to their geographically identified population. Cross sectional study was conducted among women with 0-5 year-old children (756 women) living in the area served by Number 1 PHCC. Sample size was calculated to be 255 (with 50% prevalence rate and CI: 95% SE: 5%) together with 20% substitutes, the target population was 306. The sample which was randomly stratified by midwife-area was selected from the database of PHCC. Each midwife-area is a geographically determined area with 2500-3000 inhabitants. The areas represent a socioeconomical distribution as well.

Face-to-face interviews were conducted by the researchers between January and March 2008 at the home of the participants with 83.7% response rate (n = 256). No statistically significant difference has been observed between midwife-areas in the comparison of the response rates.

### Variables

#### Maternal smoking

The women were grouped into two categories according to their smoking history during their last pregnancy.

1. Did not smoke during pregnancy: Women who did not smoke all through pregnancy. This group was subdivided into two categories: a) Non-smoker: These women were non-smokers before their pregnancy and did not smoke during pregnancy. Women who had once smoked but non-smokers at the onset of pregnancy were also included. b) Stopped smoking: These women were smokers (smoked at least 100 cigarettes in their lives) before pregnancy but stopped at the onset of pregnancy.

2. Smoked during pregnancy: Considering the dose-response relation for perinatal outcomes, women who smoked during pregnancy have been classified into two groups according to frequency of consumption: a) Occasional smoker: These women smoked during pregnancy but the consumption was not daily. b) Daily smoker: These women smoked daily during pregnancy.

#### Smoking status of partner during pregnancy

The women reported the smoking status of their partner during their pregnancy. The partners were categorized into "smoked" and "did not smoke"

#### Demographics

Maternal age, education, number of previous births, place of origin, migration, and partner's education was questioned. Education was grouped according to the educational achievements: completion of primary school, secondary school and university. Those who had not completed primary school education but could read and write were grouped as "only literate". Those who could neither read nor write were grouped as "illiterate".

The migration described in this paper is an interprovincial migration. The women were asked about their place of origin and for any migration history. If they were not born in Burhaniye but had migrated to it, they were considered as migrants.

#### Socioeconomic status

Turkish Statistical Institute (TSI) advises a poverty threshold of a daily income of 4.3$ per person [12]. Money earned or the earnings from other revenues were added to determine the household income. This was situated according to the determined limit: below or above the poverty level. The respondents were also asked how they perceive their welfare levels: Very good, good, moderate, low, very low. In analysis, the first two and last two categories were combined. Class position was determined according to the family head [13]. The categorization was based on the possession to the means of production and for this aim a class scheme developed by Boratav [14] was used.

### Statistical analysis

For the calculation of prevalences and 95% CI, Epi 6.0 was used. The relation of "smoking during pregnancy" and "daily smoking during pregnancy" with the independent variables was determined with χ^2 ^tests. In univariate and multivariate analysis, daily smokers were compared with the group of non-smokers, stopped smoking and occasional smokers during pregnancy. Statistical significance is achieved when the *p *value is less than 0.05. The variables in relation with the dependent variables in the hypothesis tests were included in the forward-stepwise logistic analysis. Variables with p-values lower than 0.05 were added to the model one at a time.

The multivariate logistic regression analysis for "Did not smoke versus daily and occasional smokers" included maternal age, maternal education, place of origin, migration, poverty, perceived income and partner's smoking status.

The multivariate logistic regression analysis for "Daily smokers versus the group of non-smokers, stopped smoking and occasional smokers" included, maternal education, social class, migration, poverty, perceived income and partner's smoking status.

### Ethical approval

Permission to use the official data of PHCC and to perform the field study was approved by the Regional Office of the Turkish Ministry of Health. This is a formal procedure that includes ethical evaluation within the Regional Office. Oral informed consent was sought from every woman within the study population.

## Results

In this study, 256 women who had a delivery in the last five years were investigated about their smoking status before and during pregnancy.

### Characteristics of the sample

The mean maternal age was 25.3 ± 5.3. The mean number of years after pregnancy was 2.83 ± 1.52. The proportion of women who had completed primary school education was 64.3%. The combination of illeterate or only literate group summed to 7.5%. 41.8% had migrated to Burhaniye.

### Prevalance of smoking

**Figure 1:**
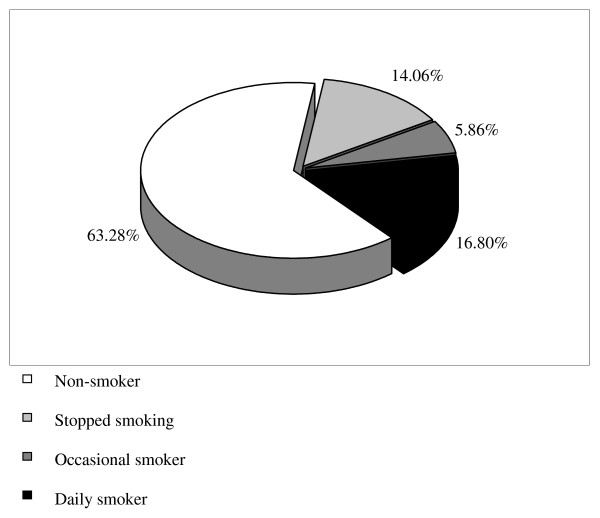
**Distribution of the smoking habits among women during pregnancy**.

The distribution of the smoking habits during pregnancy is presented in Figure 1. 63.28% (95% CI: 57.24-69.02) of women were non-smokers before pregnancy. Among the smokers, 38.30% (95% CI: 28.89-48.41) stopped smoking during pregnancy. Prevalance of smoking during pregnancy was 22.66% (95% CI: 17.84-28.09). The majority (74.14% with 95% CI: 61.76-84.15) were daily smokers. The mean number of daily cigarette consumption of the daily smokers was 9.22 ± 10.3 (median = 5). The mean number of cigarette consumption per week among the occasional smokers was 2.13 ± 1.85 (median = 1).

Table 1 displays a crosstabulation of maternal smoking during pregnancy by sociodemographic characteristics of the women.

**Table 1:** Smoking habits during pregnancy according to sociodemographic characteristics (n = 256)

		**Did not smoke during pregnancy**		**Smoked during pregnancy**	
		
		**Non-smoker (n = 162)**	**Stopped smoking (n = 36)**	**Occasional smoker (n = 15)**	**Daily smoker (n = 43)**
	**Total(n)**	**%**	**%**	**%**	**%**
	
**Maternal Age**					
< 20	37	54.1	8.1	10.8	27.0
20-34	201	64.7	15.9	5.5	13.9
≥ 35	17	70.6	-	-	29.4
*Missing*	*1*				
**Maternal education**					
Illeterate +Only literate	19	42.1	5.3	-	52.6
Primary	137	67.9	15.3	5.1	11.7
Secondary+University	99	61.6	13.1	8.1	17.2
*Missing*	*1*				
**Partner's education**					
Illeterate/Only literate	12	16.7	8.3	-	75.0
Primary	99	69.7	13.1	3.0	14.1
Secondary+University	143	63.3	14.7	8.4	13.3
*Missing*	*2*				
**Previous Births**					
Nullipar	107	62.6	15.0	9.3	13.1
1+	149	63.8	13.4	3.4	19.5
**Social class**					
Employer+Self Employed	73	67.1	15.1	8.2	9.6
White-collar employees	50	56.0	20.0	10.0	14.0
Blue-collar workers	92	68.5	12.0	3.3	16.3
Marginal, unemployed	40	52.5	10.0	2.5	35.0
*Missing*	*1*				
**Place of origin**					
Urban	47	46.8	17.0	14.9	21.3
Rural	206	67.5	13.1	3.9	15.5
*Missing*	*3*				
**Migrated to Burhaniye**					
Yes	107	54.2	15.0	6.5	24.3
No	149	69.8	13.4	5.4	11.4
**Poverty**					
Below poverty level	91	62.6	11.0	1.1	25.3
Above poverty level	165	63.6	15.8	8.5	12.1
**Perceived Income**					
Very good/Good	73	61.6	21.9	8.2	8.2
Moderate	127	68.5	11.0	5.5	15.0
Low/Very low	55	54.6	10.9	3.6	30.9
*Missing*	*1*				
**Partner's smoking status**					
Smoked	125	51.2	20.8	5.6	22.4
Did not smoke	131	74.8	7.6	6.1	11.5

### Factors associated with smoking

#### Univariate analysis

"Did not smoke" versus "daily and occasional smokers". Young mothers (less than 20), low educated parents, low social classes, migrants, those below the poverty line, women with urban birthplace and women whose partners smoked during pregnancy had significantly higher rates of smoking during pregnancy (p < 0.05).

"Daily smokers" versus the group of "non smokers, stopped smoking and occasional smokers". Low educated parents, those with low income perception, unemployed, low social classes and migrants were found significantly associated with daily smoking (p < 0.05). Table 2 summarizes the factors associated with smoking during pregnancy and daily smoking during pregnancy.

**Table 2:** Factors associated with smoking during pregnancy and daily smoking

	SMOKING STATUS					
	**Smoking during pregnancy**			**Daily smoking**		
	
	**Daily and occasional smoker** **n(%)**	**Did not smoke** **n(%)**	**p**	**Daily smoker** **n(%)**	**Non smoker, stopped smoking, occasional smoker** **n(%)**	**p**
	
**Maternal Age**						
< 20	14(37.8)	23(62.2)	0.039	15(40.5)	22(59.5)	0.215
20-34	39(19.4)	162(80.6)		53(26.4)	148(73.6)	
≥ 35	5(29.4)	12(70.6)		5(29.4)	12(70.6)	
**Maternal education**						
Illeterate+only literate	10(52.6)	9(47.4)	0.002	10(52.6)	9(47.4)	0.000
Primary	23(16.8)	114(83.2)		16(11.7)	121(88.3)	
Secondary+University	25(25.3)	74(74.7)		17(17.2)	82(82.8)	
**Partner's education**						
Illeterate+only literate	9(75.0)	3(25.0)	0.000	9(75.0)	3(25.0)	0.000
Primary	17(17.2)	82(82.8)		14(14.1)	85(85.9)	
Secondary+University	31(21.7)	112(78.3)		19(13.3)	124(86.7)	
**Previous births**						
Nullipar	23(21.5)	84(78.5)	0.802	14(13.1)	93(86.9)	0.178
1+	34(22.8)	115(77.2)		29(19.5)	120(80.5)	
**Social class**						
Employer+Self Employed	13(17.8)	60(82.2)	0.088	7(9.6)	66(90.4)	0.006
White-collar employees	12(24.0)	38(76.0)		7(14.0)	43(86.0)	
Blue-collar workers	18(19.6)	74(80.4)		15(16.3)	77(83.7)	
Marginal, unemployed	15(37.5)	25(62.5)		14(35.0)	26(65.0)	
**Place of origin**						
Urban	17(36.2)	30(63.8)	0.013	10(21.3)	37(78.7)	0.339
Rural	40(19.4)	166(80.6)		32(15.5)	174(84.5)	
**Migrated to Burhaniye**						
Yes	33(30.8)	74(69.2)	0.008	26(24.3)	81(75.7)	0.007
No	25(16.8)	124(83.2)		17(11.4)	132(88.6)	
**Poverty**						
Below poverty level	24(26.4)	67(73.6)	0.000	23(25.3)	68(74.7)	0.007
Above poverty level	34(20.6)	131(79.4)		20(12.1)	145(87.9)	
**Perceived Income**						
Very good/good	12(16.4)	61(83.6)	0.039	6(8.2)	67(91.8)	0.002
Moderate	26(20.5)	101(79.5)		19(15.0)	108(85.0)	
Low/Very low	19(34.5)	36(65.5)		17(30.9)	38(69.1)	
**Partner's smoking status**						
Smoked	35(28.0)	90(72.0)	0.046	28(22.4)	97(77.6)	0.019
Did not smoke	23(17.6)	108(82.4)		15(11.5)	116(88.5)	

#### Multivariate analysis

"Did not smoke" versus "daily and occasional smokers". This analysis presents three variables in the resultant equation; maternal age, maternal education and migration. Young women (< 20) are 3.4 times more likely to smoke during pregnancy. Illiterate/only literate women are 3.8 times and migrant women are 2.7 times at increased risk for smoking during pregnancy (Table 3).

**Table 3:** Multivariate logistic regression results for two logistic models*

	**OR (95% CI)**
	
**"Did not smoke" versus "daily and occasional smoker"**	
Maternal age	
< 20	3.41 (1.40-8.35)**
20-35	1.00
≥ 35	1.15 (0.27-4.86)
Maternal education	
Illiterate/only literate	3.79 (1.14-12.55)**
Primary education	0.79 (0.38-1.65) 1.00
Secondary/University	
Migration	
Migrated	2.68 (1.31-5.48)**
Did not migrate	1.00
**"Daily smokers" versus the group of "non smoker, stopped smoking and occasional smoker"**	
Maternal education	
Illiterate/only literate	6.79 (2.03-22.77)**
Primary education	0.91 (0.46-1.81)
Secondary/University	1.00
Migration	
Migrated	2.16 (1.12-4.15)**
Did not migrate	1.00

* The two logistic models are: 1. Did not smoke versus daily and occasional smoker and 2. Daily smokers versus the group of non smoker, stopped smoking and occasional smoker.

** p < 0,05

"Daily smokers versus the group of "non smokers, stopped smoking and occasional smokers,". Two variables have appeared in the resultant equation; maternal education and migration. Low educated women are 6.8 times and migrant women are 2.2 times at increased risk for smoking daily (Table 3).

## Discussion

In this study, smoking prevalance during pregnancy was 22.7% and this is higher than the country prevalence. Young mothers (< 20), low educated women and migrants were at increased risk for smoking during pregnancy. Low education and being a migrant were risk factors for daily consumption.

In this study the retrospective assessment has been 2.8 years after pregnancy approximately. Recall bias has been questioned in previous studies, because the recollection of events and behaviors with recall bias is a serious threat to the use of retrospective data. However, considering smoking behavior in pregnancy, recall accuracy was ascertained in studies even five or six years after pregnancy [15]. The saliency of pregnancy as a life event and the social stigmatism associated with smoking during pregnancy is assumed to create this accuracy. Thus, in this study, the 2.8 years after pregnancy should not be considered as questionable for validity. Self-report is a limitation of this study. However, 2008 systematic review on smoking during pregnancy, presents self-report as the main limitation of all studies included in the review and addresses social desirability as an important source of bias resulting in underreporting of actual prevalence [1]. This study, tried to diminish the social desirability bias. Burhaniye was a region where home visits were an essential part of primary care and midwives working at the area knew most of the women with their names. Provider sincerity was accomplished at the area, long before our study was executed; this was an important point in reducing the social desirability bias. As the research team visited the houses, they presented themselves as coming via the primary care center and that the midwives knew about this visit. This increased the response rate as well as the sincerity of the interview. The information elicited from the women at their homes rather than a health care setting and the fact that face-to-face interview was used rather than a self-administrated questionnaire made the information more reliable and thus pointed out the socioeconomic determinants more truly.

In DHS Turkey 2003, 15% of pregnant women report that they smoke regularly and among these pregnant women, 41% smoke 3-5 cigarettes, 14% smoke 6-9 cigarettes, and 15% smoke 10 or more cigarettes [6]. The high prevalence of smoking during pregnancy in this study may be attributed to the urban-rural characteristics of the town as well as its location in the Western region of Turkey where smoking prevalence of women is 32.3% [6]. Among the smoker group 38.7% quitted during pregnancy which is in accordance with studies reporting 20-40% of quitting during pregnancy [2].

Studies have shown that smoking prevalence during pregnancy is determined by social factors, especially educational level and social class [9,16]. The influence of social class on child and mother health is exerted through intermediate factors. Maternal age has been declared as one of these intermediate factors [17]. In this study, smoking was more prevalent among the young mothers. The increased risk for smoking during pregnancy for this risky age group has been 3.4 fold. The very young mothers are considered to be at higher risk for perinatal outcomes [18]. The risks of smoking during pregnancy add and make a double burden for this risky age group [2]. Very young women are shown to be more likely to smoke during pregnancy. They are probably unaware of the health related consequences of their tobacco use [19]. Maternal age becomes an intermediate determinant [6] where it serves as a media for the "causes of the causes" for cigarette smoking during pregnancy [7]. The young mothers and those of low socioeconomic status all have one thing in common: on average they have to cope with more stress, less favorable social networks and poorer economic conditions. All these make it harder to quit smoking during pregnancy because smoking is a practice which promises stres relief, albeit short-lived [1,20]. Smoking enhances the sense of well being and is used as a tool to cope with negative mood or stress experiences [21]. It has been declared that half the excess risk of adverse perinatal outcomes in the lowest socioeconomic group is explained by maternal smoking [11], so the efforts aiming to reduce smoking during pregnancy especially among the deprived groups is an important intervention for perinatal health.

In this study, in univariate analysis partner's smoking status was significantly associated with maternal smoking during pregnancy. Smoking status of partner is a well documented determinant of maternal smoking during pregnancy [22]. Smokers are less likely to give up the habit if others smoked daily at home [3].

An individual's probability of smoking is independently associated with a vast array of different indicators of social disadvantage. Any marker of disadvantage that can be envisaged and measured whether personal, material or cultural is likely to have an independent association with cigarette smoking [20]. Migrants in our population can be considered as those having this socioeconomic disadvantage and they were shown to have a higher prevalance of smoking during pregnancy. Internal migration has had a great impact on Turkey's population dynamics for decades. According to the 2000 population census, nearly 28% of the population was born in a different province that they now reside in. It is generally a result of a transfer of labor from low productive to high productive areas [23]. In this sense, Burhaniye has been an attractive town with its well known place in farming, olive oil industry and tourism. However, Ozmucur and Silber showed that internal migration from rural to urban areas increased the income inequalities rather than acting as an equilibrating mechanism and closing the gap [24]. Thus, the internal migrants can be described as disadvantageous groups of the population, especially at urban settings. The multivariate analysis showed that migrants were at increased risk for smoking during pregnancy as well as an increased risk for smoking daily. Those who were born in the urban were more likely to smoke. This may be attributed to the higher prevalances of cigarette smoking for women in urban areas rather than an increased smoking prevalance in pregnancy. Smoking prevalance of women in Turkey is higher in urban areas (32.8%) than in rural (14.9%) [6]. Women originating from urban places were culturally more welcome to smoking than those originating from rural settings.

The number of cigarettes the mother smokes during pregnancy increases the perinatal risk [25], thus for this study, daily smokers are at increased risk for perinatal outcomes. Studies have shown that the risk of SGA births, preterm births and stillbirths increases with amount smoked and smoking cessation makes improvements in these risks [3]. In this study, low educated women and migrants were at increased risk for smoking daily. The daily cigarette consumption in this group with low socioeconomic conditions signals the aggravation of risks for poor fetal growth and thus unhealthy perinatal outcomes [26-28].

Smoking cessation programs need to be implemented in all maternity care settings and attention to smoking behaviour together with support for smoking cessation and relapse prevention needs to be a routine a part of antenatal care as the measurement of blood pressure [29]. Primary care services are a very important source of prenatal care especially for low income, low educated and migrant populations in developing countries [30]. This makes their role more important, in the efforts to reduce smoking during pregnancy. All primary care settings are of great importance. Considering that, implementation should be at all settings, home visits by midwives deserve special focus. Home visits are an important part of the comprehensive prenatal care services that entail outreach efforts to improve enrollment in prenatal care. This is especially important for low income populations [31]. Moreover, gender-based barriers to utilization, especially restrictions that prevent women from leaving their homes to access health services on their own are thus overcome. The conception of health systems as core social institutions moves the analysis beyond a simplistic view of healthcare as a technical, biomedical fix to a recognition that both health and healthcare are deeply embedded in broader webs of social and economic forces. Priority must be given to primary care facilities and to community-based primary care activities, often linked to the health care facilities, especially when they truly empower the communities they serve [32]. Moreover, the unreliability of self-report as a measure of smoking status in the health care settings [29] may also be overcome at the home of the women with more sincere atmosphere shared with her midwife. For high income countries the most effective intervention has been reported to be providing incentives. Not much is known about the development or implementation of such interventions or their effectiveness in low or middle income countries [33]. For Turkey, further research is necessary in evaluating and comparing the effectiveness of such programmes in the light of the focus on social inequalities presented in this paper.

Studies show that, women who stop smoking in early pregnancy had birthweights essentially the same as nonsmokers and the risk of stillbirth also reduced to that of nonsmokers [34,35]. Considering the effect of smoking on adverse perinatal outcomes, the importance of high smoking prevalence of pregnant women of low socioeconomic conditions presented in this study becomes more significant. Turkey is a country with high levels of infant mortality and the rate increases dramatically for groups with low education and low socioeconomic status [36]. For these groups, the unhealthy living conditions, bariers for access to heath care and incapacity of the health care system for access to obstetric emergency services may combine with the perinatal risks of smoking during pregnancy. Smoking during pregnancy fuels inequities in perinatal outcomes. To reduce inequalities later in life it is important to reduce inequalities at birth [37].

### Study limitations

Information on smoking was elicited from the women themselves. Self reported information of smoking prevalance during pregnancy is probably underestimated.

## Conclusions

Systematic attention should be paid to socioeconomic determinants in smoking for pregnant women. This is vital, especially for countries like Turkey, with high rates of infant and mother mortality and substantial health inequalities. Young maternal age groups, low educated women and migrants are important groups to focus on. Acknowledging smoking as a major source of social differences in health and adressing them even in intrauterine life will help to design better smoking intervention programs and to develop opportunities for better perinatal health.

## Competing interests

The authors declare that they have no competing interests.

## Authors' contributions

IE, HH and FAT have made substantial contributions to the conception and design of the study, IE, HH and GA contributed to the acquisition of data; IE, HH and FAT have made significant contributions to the analysis and interpretation of the data; IE, HH and GA were involved in drafting the manuscript and revising it critically for important intellectual content; and IE and HH gave final approval of the version to be published. All authors read and approved the final manuscript.

## Pre-publication history

The pre-publication history for this paper can be accessed here:

http://www.biomedcentral.com/1471-2458/10/325/prepub
